# Behavioral responses of a parasitoid fly to rapidly evolving host signals

**DOI:** 10.1002/ece3.9193

**Published:** 2022-08-11

**Authors:** E. Dale Broder, James H. Gallagher, Aaron W. Wikle, Cameron P. Venable, David M. Zonana, Spencer J. Ingley, Tanner C. Smith, Robin M. Tinghitella

**Affiliations:** ^1^ Department of Biology University of Denver Denver Colorado USA; ^2^ Brigham Young University–Hawaii Laie Hawaii USA; ^3^ Brigham Young University Provo Utah USA

**Keywords:** novelty, *Ormia ochracea*, parasite–host, phonotaxis, preference, *Teleogryllus oceanicus*

## Abstract

Animals eavesdrop on signals and cues generated by prey, predators, hosts, parasites, competing species, and conspecifics, and the conspicuousness of sexual signals makes them particularly susceptible. Yet, when sexual signals evolve, most attention is paid to impacts on intended receivers (potential mates) rather than fitness consequences for eavesdroppers. Using the rapidly evolving interaction between the Pacific field cricket, *Teleogryllus oceanicus*, and the parasitoid fly, *Ormia ochracea*, we asked how parasitoids initially respond to novel changes in host signals. We recently discovered a novel sexual signal, purring song, in Hawaiian populations of *T. oceanicus* that appears to have evolved because it protects the cricket from the parasitoid while still allowing males to attract female crickets for mating. In Hawaii, there are no known alternative hosts for the parasitoid, so we would expect flies to be under selection to detect and attend to the new purring song. We used complementary field and laboratory phonotaxis experiments to test fly responses to purring songs that varied in many dimensions, as well as to ancestral song. We found that flies strongly prefer ancestral song over purring songs in both the field and the lab, but we caught more flies to purring songs in the field than reported in previous work, indicating that flies may be exerting some selective pressure on the novel song. When played at realistic amplitudes, we found no preferences–flies responded equally to all purrs that varied in frequency, broadbandedness, and temporal measures. However, our lab experiment did reveal the first evidence of preference for purring song amplitude, as flies were more attracted to purrs played at amplitudes greater than naturally occurring purring songs. As purring becomes more common throughout Hawaii, flies that can use purring song to locate hosts should be favored by selection and increase in frequency.

## Graphical abstract

When host signals change, the effects on eavesdroppers are often ignored. We used the rapidly evolving interaction between the Pacific field cricket, *Teleogryllus oceanicus*, and the parasitoid fly, *Ormia ochracea*, to ask how parasitoids initially respond to a novel host signal (purring). We found fly preferences for amplitude but no other components (e.g., frequency, temporal) of novel purring song. As purring spreads throughout Hawaii, we expect selection to favor flies that use purring song to locate their hosts.

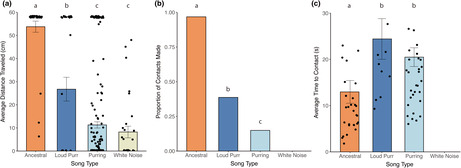

## 1 INTRODUCTION

Animals communicate through many modes (e.g., acoustic, visual, and chemical) to convey diverse and often complex information with conspecifics (e.g, mate quality or receptivity, competitive ability) and heterospecifics (e.g., unpalatability). Because the purpose of many signals is to get the attention of receivers, signaling traits are often conspicuous, making them subject to multiple different, and sometimes conflicting, selective pressures (Darwin, 1871; Zuk & Kolluru, 1998). For instance, animals eavesdrop on signals and cues generated by prey, predators, hosts, parasites, competing species, and conspecifics (potential mates or competitors). While eavesdroppers are not the intended receivers of these signals, they have the potential to shape the evolution of such traits (Belwood & Morris, 1987; Bertram et al., 2004; Endler, 1987). Evolutionary changes in signals likely also affect unintended receivers, and the impact may depend on the nature of the relationship between the signaler and the eavesdropper. For example, failure to attend to changed cues and signals could have drastic fitness consequences for animals who rely solely on eavesdropping to avoid predation or to find food, mates, or hosts, especially when no alternatives are available. Such a situation would lead to strong selection to attend to local cues and signals (Kaltz & Shykoff, 1998) and may prompt shifts in eavesdropper behavior in response to changes in local signals (Page & Ryan, 2005). However, while tightly coupled species could increase the selection pressure on the eavesdropper to adapt accordingly, the same relationship might make it more likely that host signals evolve to escape future eavesdropper pressure. Parasitoids (parasites that kill their host; Eggleton & Belshaw, 1992) are tightly coupled to their hosts as they are dependent on finding suitable hosts (often via eavesdropping; Lloyd & Wing, 1983; Vinson, 1976) in which to deposit their eggs or larvae. Here we capitalize on a rapidly evolving interaction between the Pacific field cricket, *Teleogryllus oceanicus* (hereafter “cricket”), and the acoustically orienting parasitoid fly *Ormia ochracea* (hereafter “fly” [Cade, 1975]), to understand how parasitoids initially respond to novel changes in host sexual signals.

We recently discovered a novel sexual signal, purring song, in Hawaiian populations of *T. oceanicus* that appears to have evolved to protect singing male crickets from the parasitoid fly while still allowing them to attract female crickets for mating (Tinghitella et al., 2018, 2021). In Hawaii there are no known alternative hosts for the parasitoid fly (Heinen‐Kay & Zuk, 2019; Otte, 1994), so we would expect flies to be under strong selection to detect and attend to the new purring song (note that selection to attend to purring songs would be lower if ancestral *T. oceanicus* males are present in a given population). Because this work focuses on Hawaii where there is only one host, we use “ancestral” to refer to the ancestral *T. oceanicus* male morph throughout this manuscript, but note that the ancestral cricket host species of *O. ochracea* are hypothesized to be *Gryllus rubens* and *Gryllus texensis* (Gray et al., 2019). While the ancestral *T. oceanicus* song is relatively loud and nearly pure tone with a peak frequency of 4.8 kHz, the purring song is more broadband, quieter, higher in median peak frequency, and more variable among males (Tinghitella et al., 2018), all of which should affect detectability by the flies (Oshinsky & Hoy, 2002; Robert et al., 1992). Indeed, in extensive field studies we found that only a single parasitoid fly (of 48 captured in sound traps) successfully located a purring song, suggesting purring is a private mode of communication among crickets (Tinghitella et al., 2021). However, we find *O. ochracea* in locations where no ancestral males are present, and in controlled laboratory settings, some flies are able to locate some purrs at short distances (Tinghitella et al., 2021). The recent evolution of purring song thus provides a rare opportunity to study how parasitoid flies respond to a novel host signal soon after its origin.

We conducted two complementary experiments, field and lab, both of which aimed to compare fly responses to purring versus ancestral song as well as capture fly responses to the multidimensional variation in the recently evolved purring cricket songs. The field experiment involved playing variable purring songs and ancestral song simultaneously under natural conditions (ambient anthropogenic and environmental sound; ancestral, purring, and silent morphs present in the population) where there are competitive signalers and flies have choices. This experiment reveals the selection exerted by a wild fly population in situ. Given that flies persist in populations devoid of ancestral crickets and are able to locate some purring songs at close distances, we then played the same set of variable songs to individual flies in no‐choice host location experiments in a controlled laboratory setting. Because we played all song stimuli to each fly, this experiment had a larger sample size and allowed us to measure individual fly preferences. This experiment mimicked a situation in which ancestral males were absent and captured the selection the flies would exert on purring song variation under “ideal” circumstances devoid of competing signalers and environmental noise, and allows us to make predictions about possible future evolution of cricket song in response to flies.

In both the field and lab host location experiments, we first asked about natural selection (preferences) imposed by flies across song types (ancestral vs. purring vs. a white noise control) and then characterized preferences for purring variants (selection acting on different purrs) by comparing fly attraction to eight purring songs that capture the multivariate natural variation in song. For the field experiment we predicted first that flies would prefer ancestral song over purring song, but would not prefer purring song over white noise, supporting the idea that purring is a private mode of communication (Tinghitella et al., 2021). However, previous work only broadcast songs at biologically realistic volumes, and purring song is quieter than ancestral song (Tinghitella et al., 2021). Amplitude could help explain the preference for ancestral over purring song, so we additionally played one purring song at the same amplitude as the ancestral song stimulus. Identical responses to the ancestral and the loud purr stimuli would indicate that amplitude explains much of fly responses. Alternatively, if there is no natural selection pressure from flies keeping amplitude of purring songs in check (no difference in response to loud purr vs. natural purrs), purring songs may become louder over time if female crickets prefer songs with greater amplitude. If our first prediction is correct, we may capture few flies at purring traps, reducing our power to test for preferences among purring variants. We can address this limitation through lab experiments that expose individual flies to each song.

As with the field experiment, in the lab experiment we first asked about selection by flies across song types and then asked about preferences for variants of purring song. Because some flies could locate some purrs over short distances in previous work (Tinghitella et al., 2021), we hypothesized that flies would be most attracted to ancestral song but would prefer purring song over white noise in the lab. We also expected the response to the higher amplitude purr (vs. realistic amplitude purr) to match our findings in the field. The design of the lab experiment allowed us to generate preference functions for each individual fly and to test two alternative hypotheses about the form of population‐level preference functions (selection) acting on different purring variants. The first possible hypothesis is that flies have no preference for particular purrs (preference functions are flat). This result seems likely for three reasons: (1) preference evolution may lag behind signal evolution (Broder et al., 2021; Rosenthal, 2018), (2) flies may be pre‐adapted to broadly attend to any new host signals because they parasitize at least 17 other cricket species in other parts of their range (Gray et al., 2019), and (3) there is a large fitness cost for flies that cannot locate a host, so parasitoids should accept any suitable host (Kruitwagen et al., 2021). Alternatively, flies may exhibit preferences for certain purring songs. One mechanism that could explain the existence of preferences immediately upon the origin of a new host signal is sensory biases (Ryan et al., 1990); flies may prefer songs that have spectral characteristics aligned with their sensory system. For example, *O. ochracea* ears are tightly tuned to detect songs with a peak frequency around 4.9 kHz (Oshinsky & Hoy, 2002; Robert et al., 1992). Finally, we expect higher response rates in the lab than in the field because, in the lab, the distance between song stimulus and fly is short, there are no competing songs, and there is no background noise. A detailed understanding of the selection imposed by flies among and within novel cricket songs is critical to predict the evolutionary trajectory of the novel signal as well as the demographic and evolutionary outcomes for fly populations.

## 2 METHODS

Both field and lab experiments were conducted between June 2019 and July 2021 in Laie, HI using the population of flies present at Brigham Young University–Hawaii. This population contains silent (also called flatwing; Pascoal et al., 2014), purring, and ancestral crickets as well as a high density of flies (Tinghitella et al., 2021). We conducted the field trapping experiment and the lab phonotaxis experiment during the active period of *O. ochracea* (Cade et al., 1996); from approximately 1 h before sunset to 1 h after; ~6–8 p.m. HST.

For both the field and laboratory experiments, we played the same set of songs: eight purring variants, one loud purr, one ancestral song, and a white noise negative control. The same set of songs (except for loud purr) were used in a previous study that examined female cricket preferences for purring songs (Tinghitella et al., 2021). Briefly, to choose purring exemplars, we analyzed natural purring song recordings from 46 males and measured 11 acoustic characteristics, then reduced these variables to two PC axes that together explain more than 50% of the frequency, bandwidth, and temporal variation in calling song. PC1 largely captured frequency components of song and PC2 largely captured measures of broadbandedness (e.g., number of frequency peaks and the range of frequency bands present at a standardized amplitude; for full list of acoustic measures used, see Tinghitella et al., 2021). We selected eight purring exemplar songs that captured the full range of variation in songs across purring acoustic space (see detailed methods in Tinghitella et al., 2021). For the loud purr, we randomly selected one exemplar from among the four most central exemplars in principal component space, which we played at a louder amplitude (same as the ancestral song). For the ancestral song, we also used naturally recorded songs from animals in our populations, but we combined songs from four males, which we looped to avoid pseudoreplication. To standardize the amplitudes during playbacks, we digitally adjusted the amplitudes (RMS level) of all stimuli in Logic Pro X, as was done in previous work (Tinghitella et al., 2021). For both the field and lab experiments, we broadcast stimuli at 70 dBA (at 1 m) for ancestral and “loud purr” (biologically realistic volumes for ancestral) and 53 dBA for all purring stimuli and white noise (realistic amplitude for purring).

### 2.1 Field host location experiment

To quantify the selection exerted by flies on cricket song in a natural field setting, we used funnel trap arrays (following Walker, 1989) to broadcast the 11 songs described above. Each funnel trap was fashioned from a clear 2‐L plastic bottle and contained a speaker (AGPTEK A02 MP3 player that has an internal speaker) that broadcast stimuli as in Tinghitella et al. (2021). This experiment was conducted by students in an animal behavior CURE (Course‐based Undergraduate Research Experience) at BYU–Hawaii led by two of the coauthors, SJI and TCS (Smith et al., 2021). In two adjacent fields on campus known to have high cricket and fly abundance, the students deployed funnel traps twice weekly throughout the fall semester (September 24 to November 26, 2019) resulting in 16 replicate trap nights (*N* = 16 ancestral, 16 white noise, 16 loud purr, and 128 purring exemplars). The traps were placed 10 m apart along a transect in a random order. After the 2‐h trapping period at dusk, students recorded the number of flies caught in each trap.

### 2.2 Lab preference experiment

We collected flies approximately every 6 months for four total time points over 2 years (June 2019–July 2021), in the same fields described above (at BYU‐Hawaii). We attempted to collect flies using traps broadcasting both ancestral and purring songs, but we only captured them at traps broadcasting ancestral song. Upon collection, we housed flies in mesh butterfly cages (40 × 40 × 61 cm, Transfit brand) with fruit juice, water, and shelter for 24 h before being used in phonotaxis experiments. Flies were kept indoors at ambient temperatures (no climate control) in partial shade and allowed to experience natural sunlight to maintain photoperiod.

To determine how flies (*N* = 31) respond to different variants of purring song at shorter distances and without competing sounds, we conducted a host location (phonotaxis) experiment indoors at ambient temperature under red light during their active period using the same 11 songs described above. All flies were individually tested with all songs in a random order except that the loud purr and ancestral song were played second‐to‐last and last, respectively, to avoid the possibility that hearing these higher amplitude songs would change responses to subsequent stimuli. To begin each trial, an individual fly was placed into an empty butterfly mesh cage (40 × 40 × 61 cm) and gently directed to the top of the cage. Underneath the bottom of the mesh cage, we placed a speaker (AOMAIS Sport II) in one of four corners (speaker location randomized for each song stimuli). For each song played, two trained observers watched each trial continuously and agreed on all measures independently. We measured the vertical distance the fly traveled toward the speaker (max distance = 58 cm) using a cm measuring tape that was attached directly to the outside of the cage, whether the fly contacted the speaker (yes or no), and if they did, the time to contact (max time = 60 s). Contact (yes/no) directly estimates the natural selection exerted by flies on crickets, while distance traveled indicates a positive phonotactics response (Goodson & Adkins‐Regan 1997; Hedwig & Poulet, 2004; Mason et al., 2005; Sarmiento‐Ponce et al., 2021; Tinghitella et al., 2021). Between playbacks we gently directed flies back to the top of the cage, allowed a minimum of 5 s between stimuli, and waited until flies ceased grooming or moving before playing the next stimulus. After the experiments, all flies were immediately released at their capture site.

### 2.3 Statistical analysis

All statistics were run using R Studio (version 1.3.1073), and our R markdown is available as a supplemental file. For the field experiment, we first compared how the flies responded to the different song types as well as how selection acts on amplitude (comparing responses to ancestral, purring, loud purr, and white noise). We used a generalized linear model (GLM; family = Poisson) with song type as the independent variable (ancestral, purring, loud purr, white noise) and number of flies caught per trap as our dependent variable. In this model, we pooled the eight purring exemplars (excluding loud purr) into one purring category (hereafter purring). To examine differences in fly attraction among the four different song types, we then performed a post hoc Tukey's test (package *emmeans*; Lenth et al., 2019). To test for preferences among purring exemplars only (the shape of selection from flies acting on the novel purring signal), in the field trapping experiment, we used a second GLM with the same model structure described above except that the independent variable was the eight purring exemplar songs, rather than song type. In this model, we excluded ancestral song, white noise, and the loud purr.

In the lab phonotaxis experiment, there were two flies that did not respond to any stimulus, so we excluded them from all models (leaving *N* = 31 overall). Again, we first tested for preferences among song types and for amplitude within purring song using mixed models. We ran separate random intercept mixed models for each of the three dependent variables in the package *lme4* (Bates et al., 2014): distance traveled (linear mixed model [LMM]; family = Gaussian), contact (GLMM; family = binomial), and for those flies who contacted the speaker, time to contact (LMM; family = Gaussian). For each model, we included song type as the independent variable (ancestral, purring, loud purr, and white noise) and individual fly ID as a random effect. Finally, when appropriate (when full model was significant), we used post hoc pairwise comparison (*emmeans* package; Lenth et al., 2019) to identify differences among song types and purring exemplars from the above models.

Next, to determine whether flies exhibit preferences for certain purring song characteristics over others we removed responses to ancestral, loud purr, and white noise and compared responses to the eight purring exemplars. Because purrs vary in numerous characteristics, in previous work, we created composite variables describing the variation using principal component analysis, yielding two PCs that together explain more than 50% of the frequency, broadbandedness, and temporal variation (Tinghitella et al., 2021). We first visualized preference surfaces by plotting thin‐plate splines in package *mgcv* (Wood, 2003) using smoothing terms for PC1 and PC2 and individual fly ID as a random effect. Next, we fit two complete second‐order mixed effects models (GLMM for contact; LMM distance) in the package *lme4* (Bates et al., 2014) and a complete second‐order linear model (LM for time to contact; smaller sample sizes precluded random effects for this model). For all three models, we included the coordinates of exemplar songs along the first two PCA axes (PC1, PC2), their interaction (PC1:PC2), and their quadratic terms (PC1^2^, PC2^2^) as our predictor variables. Additionally, for the two mixed effect models, we included individual ID as a random effect to account for repeated testing of individuals. For each dependent variable, we also ran reduced models that excluded the quadratic terms and interactions, and we used AIC to compare them, choosing the simplest model with the lowest AIC value. To examine the importance of variation among individuals in response to purring exemplars, we also inspected the standard deviation of our random effects (ID) in each model; higher standard deviation values indicate more variation among individuals. We also ran generalized linear mixed models with the categorical predictor of exemplar ID, rather than their associated PCs, and found qualitatively the same results.

## 3 RESULTS

### 3.1 Field host location experiment

**FIGURE 1:**
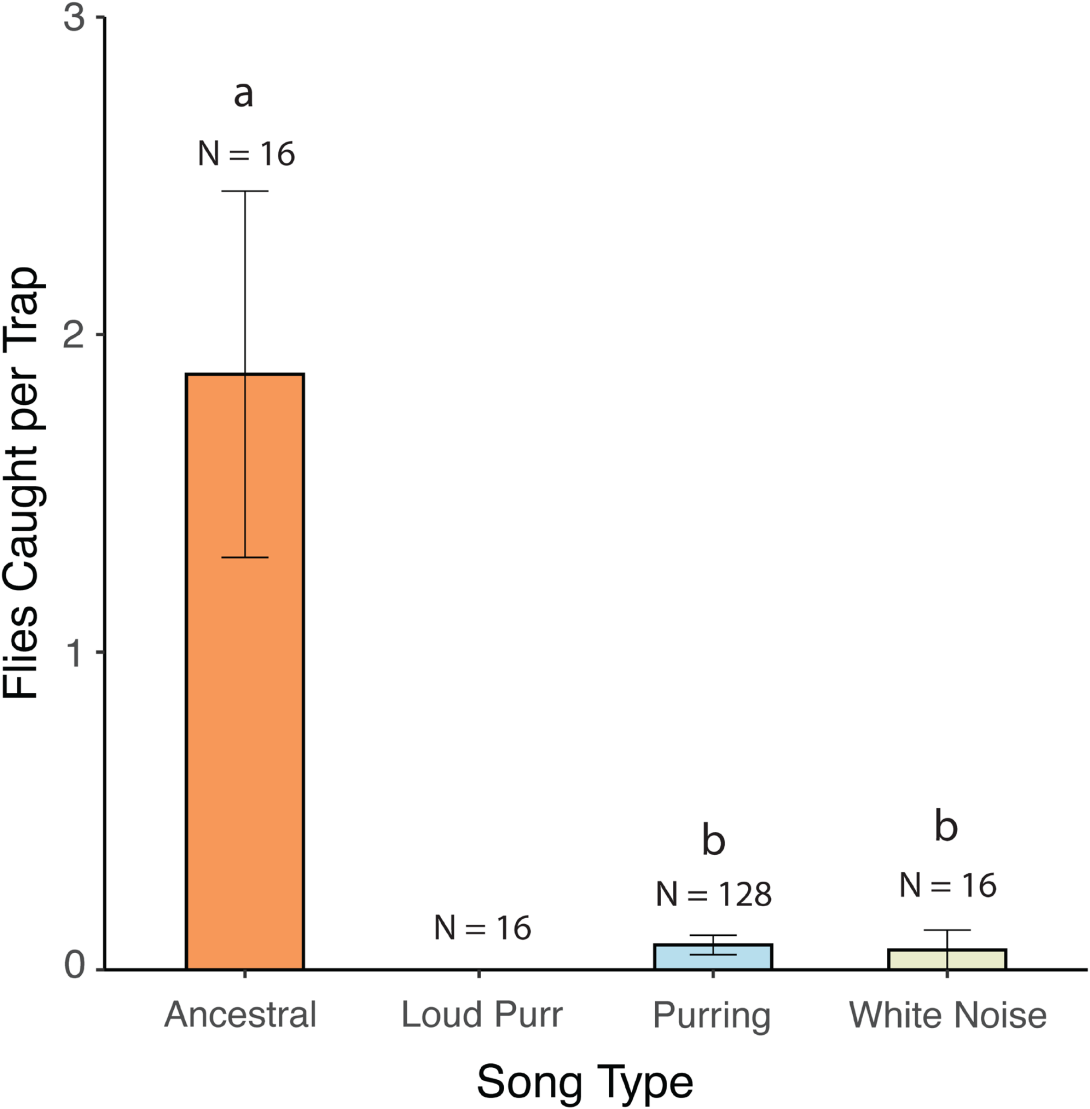
Number of flies captured per trap per night to sound traps broadcasting different song types (ancestral, loud purr, purring, white noise). Sample sizes (traps deployed) are listed above each bar. Bars with the same letter are not significantly different from one another (“loud purr” excluded from Tukey because it caught no flies). Note that there were 128 traps broadcasting purring song (eight exemplars × 16 transects) and 16 of all other stimuli (one each per transect).

In the field under semi‐natural conditions, parasitoid flies showed a strong preference for ancestral song (Type III Wald Chi square test: Χ^2^ = 84.052, df = 2, *p* < .0001; Figure 1). In pairwise comparisons, flies strongly preferred ancestral song over purring (Tukey's contrast of estimated marginal means: estimate = 3.184, *z*(155) = 8.793, *p* < .0001) and ancestral over white noise (estimate = 3.401 *z*(155) = 3.375, *p* = .0021), but there was no difference between purring and white noise (estimate = 0.217, *z*(155) = 0.209, *p* = .976). When we only considered the purring songs, we found no evidence for preferences among the different purring songs; flies were equally likely to be found in purring traps playing the eight different exemplars (Likelihood ratio Χ^2^ = 12.173, df = 7, *p* = .095). One potential explanation for capturing flies at purring traps and white noise is their proximity to traps broadcasting ancestral song. To test this idea, we calculated the mean distance (number of traps rather than linear distance) between the position in the transect of ancestral traps and those that captured flies to purring stimuli or white noise. Then we tested whether this distance was closer than expected by chance by comparing our observed mean to expected values calculated using 100,000 random permutations (we calculated a one‐sided *p*‐value as the proportion of permuted means that were equally or more extreme than our observed value;). Distance from ancestral traps did not affect the likelihood of trapping a fly at a non‐ancestral trap (*p* = .821; Figure S1). Because only 10 flies were captured in purring traps in the field, we lacked power to assess fly preferences using these data, prompting us to pursue lab‐based preference trials.

### 3.2 Lab preference experiment

**FIGURE 2:**
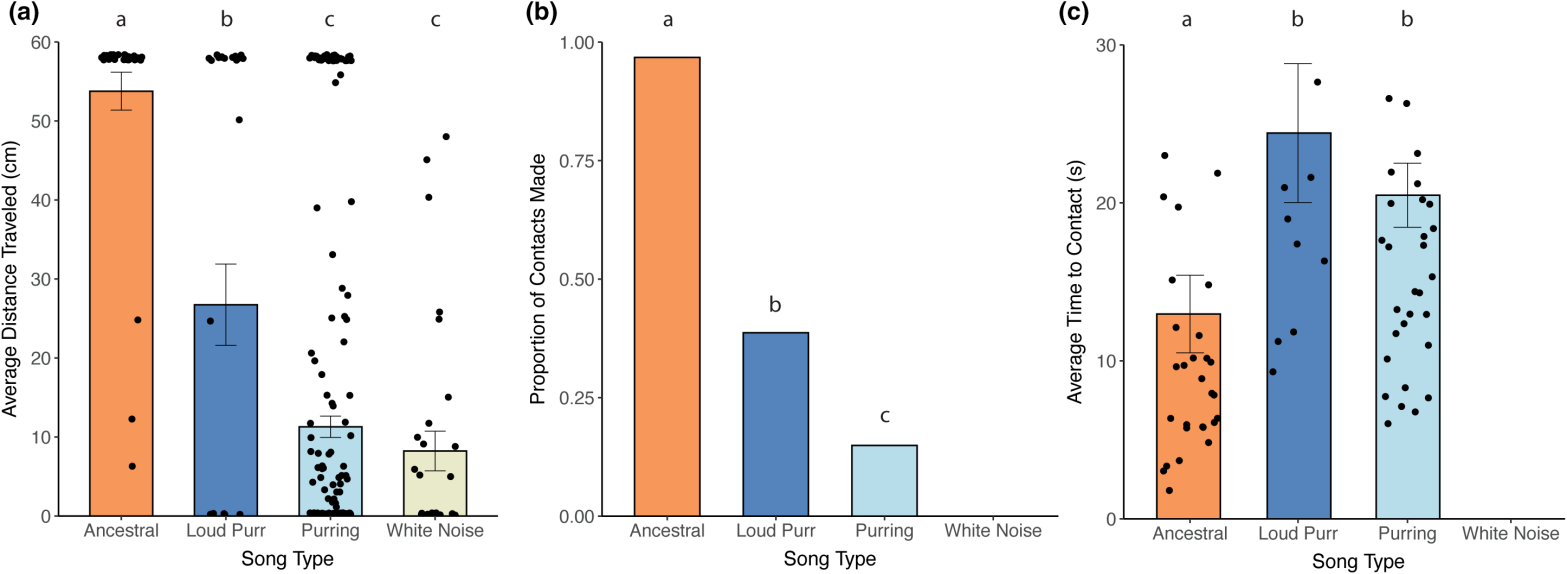
Behavioral responses to song stimuli (ancestral, loud purr, purring, white noise) in lab‐based phonotaxis tests. Positive responses were measured as (a) distance traveled toward the song stimuli, (b) proportion of flies that contacted the speaker, and (c) average time in seconds to contact the speaker for those that made contact. Bars with the same letter are not significantly different from one another (“white noise” excluded from Tukey in B and C because it caught no flies). Points in A and C represent the raw data, individual fly responses.

In the lab experiment, we first found that flies responded differently to the different song types (ancestral, loud purr, purring, and white noise) across all response variables (distance traveled: Wald Χ^2^ = 206.16, df = 3, *p* < .001; contact: Wald Χ^2^ = 30.671, df = 2, *p* < .001; time to contact: Wald Χ^2^ = 13.325, df = 2, *p* < .01). For distance traveled, flies traveled further to ancestral song than to all others (Tukey's *p* < .01) and traveled further to loud purr than purring or white noise (Tukey's *p* < .01), but responses to purring and white noise did not differ (*p* = .76; Figure 2a). For contact (yes/no), more flies contacted ancestral song followed by loud purr followed by purring (Tukey's all *p* < .01; Figure 2b). We could not include white noise in a post hoc analysis for contact or time to contact because no flies contacted white noise. For time to contact, flies contacted ancestral song faster than the loud purr (Tukey's *p* = .016) and faster than purring (Tukey's *p* = .008), but there was no difference in time to contact between the loud purr and purring (Tukey's *p* = .945; Figure 2c).

**FIGURE 3:**
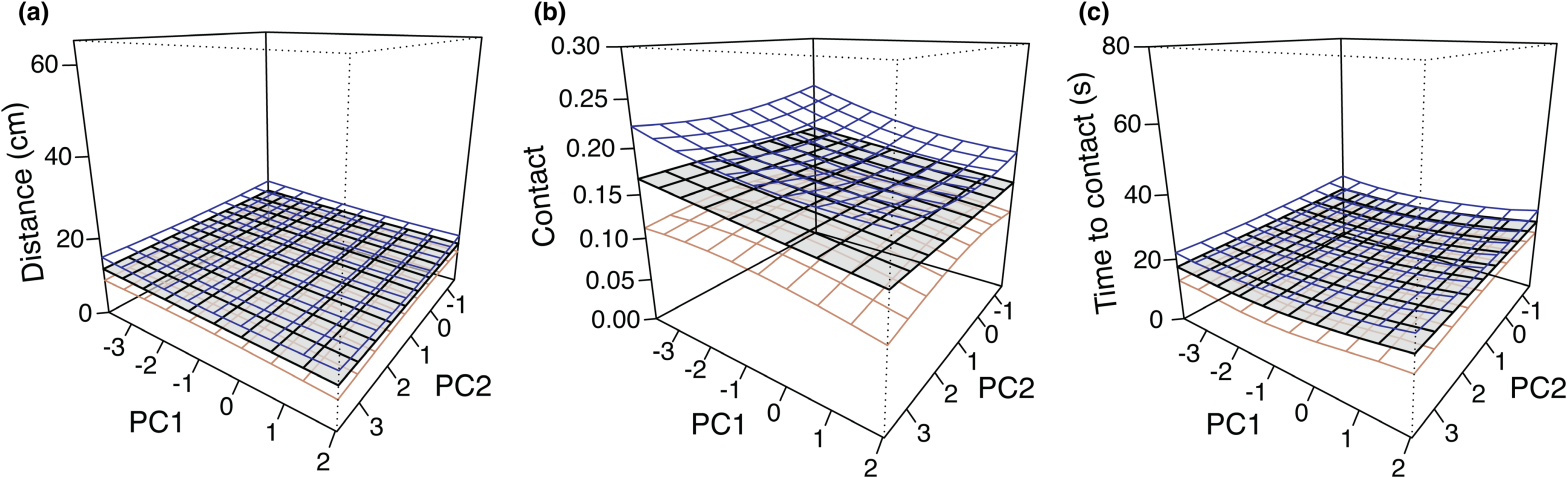
Preference surfaces describing selection exerted by parasitoid flies across the acoustic space of purring songs (exemplars). Song characteristics of the eight purring exemplars span the PC1 and PC2 axes, and fly responses to those songs are shown on the vertical axis. The gray surface shows the mean response, and the blue and orange layers show the standard error around the mean. Panel (a) shows how far the fly traveled toward the speaker broadcasting the exemplar, (b) shows the proportion of flies that contacted the speaker, and for those flies that contacted the speaker (c) shows the time (s) to contact. Note that for both distance traveled and time to contact, smaller values represent a greater response. See Table 1 for estimates of fixed and random effects.

Next, to determine whether flies exhibit preferences for certain purring song characteristics over others we removed responses to ancestral, loud purr, and white noise and visualized preference surfaces and fit mixed models describing fly responses to the PC values associated with the eight purring exemplars (natural selection imposed on purring songs). This method captures the preferences (or lack thereof) that flies have for natural variation in purring songs—in other words, the selection flies are exerting on the novel signal. For all three response variables (distance traveled, contact or not, time to contact), the simplest models containing only PC1 and PC2 as fixed effects provided the best fit (lowest AIC). Flies exhibited no significant differences in response to particular values of PC1 (frequency components of song) or PC2 (broadbandedness measures; Table 1) resulting in flat preference surfaces (Figure 3). As in previous work with *T. oceanicus* (Tinghitella et al., 2021), we found quite a lot of inter‐individual variation in the responses of different flies to the purring exemplars (see the standard deviation of the random effect individual ID in Table 1). For example, for distance traveled, the standard deviation for individuals (random effect) was 15.92, which is orders of magnitude larger than the effect of PC1 (−0.356) and PC2 (−0.219). Approximately 71% (22/31) of flies did not contact any purring exemplars (this does not include loud purr); 10% (3/31) of flies contacted one exemplar; 10% (3/31) of flies contacted four purring exemplars; 10% (3/31) contacted almost all of the exemplars (seven or eight). Lastly, 23% (7/31) contacted the loud purr but none of the purring exemplars.

**TABLE 1:** Outputs of generalized linear mixed models determining fly responses to purring exemplars (principal components) in the lab‐based fly phonotaxis experiment.

Model	Fixed effects		Random effects		
Parameter	Beta	*p*‐value	*N*	Variance	SD
Distance					
*(Intercept)*	11.455	<.001			
*PC1*	−0.356	0.393			
*PC2*	−0.219	.659			
*Individual*			31	253.4	15.92
Contact (Y/N)					
*(Intercept)*	−8.053	<.001			
*PC1*	−0.125	0.382			
*PC2*	−0.014	0.934			
*Individual*			31	60.77	7.796
Time to contact					
*(Intercept)*	20.904	<.001			
*PC1*	0.759	0.429			
*PC2*	−0.284	0.807			

*Note*: Significant findings are in bold. Note that responses to ancestral song, loud purr, and white noise were removed from these models. There are no random effect statistics for the time to contact model, as this was run as a linear model.

## 4 DISCUSSION

We set out to address how parasitoids respond to a novel host signal soon after the origin of the novel signal. In summary, we found that in both a field experiment with natural background noise and a complementary laboratory experiment, flies strongly prefer ancestral song but that some flies can locate the novel purring songs (Figures 1 and 2). In the laboratory experiment, flies preferred purring songs with higher amplitude (70dBA) over purrs played at a realistic amplitude (53dBA; Figure 2). Our laboratory experiment also allowed us to test hypotheses about the shape of selection that flies impose on purring song when locating hosts. We found overall flat preference surfaces, meaning that flies responded equally to all purring variants (Figure 3). We also found incredible interindividual variation in fly responses.

As expected, flies preferred ancestral songs over novel purring songs in both the field and the lab, matching previous work (e.g., Tinghitella et al., 2021). We also found that in the lab, flies preferred, or at least had more passive attraction, to purring songs with greater amplitude over quiet purrs. This is perhaps not surprising since *Ormia ochracea* are attracted to higher amplitude songs produced by *Gryllus lineaticeps* (Wagner, 1996), and louder stimuli should elicit a stronger neural response. It is unclear whether this response is due to detectability or preference, and neurophysiology experiments will be required to answer this important question. Based on the results in this study (flies contacted loud purr more than purring exemplars), the low amplitude of the purr could partially explain how it functions as a private mode of communication among crickets (Tinghitella et al., 2021). However, flies still contacted ancestral song at a much higher rate than purring at the same amplitude (loud purr), indicating that other features of purring song (e.g., frequency or broadbandedness measures) must also explain reduced fly responses to purrs. Importantly, we caught more flies at purring traps in the field (*N* = 10/128) than in previous work (*N* = 1/80; Tinghitella et al., 2021), though it is possible this difference is due to differences in methods between studies. Tinghitella et al. (2021) played a single purring loop (made up of five male songs) in a triangle of traps with an ancestral loop and a white noise control while we played eight different purring exemplars representing much of the natural variation in purring as well as a loud purr to test effects of amplitude, and these eight purrs only competed with only one ancestral trap and one white noise trap. While ancestral song is still significantly preferred and purring is clearly protective, some flies (about 8% in our study) can locate hosts in the wild using this novel song. Because purring is currently increasing across Hawaii (unpublished), there should be strong selection favoring those flies that can locate purring songs. If the capacity of parasitoid flies to respond to purring is genetically based, we expect it to increase in frequency in the future.

We explored fly preferences for purring songs that varied in many dimensions (frequency, broadbandedness, temporal) in the lab experiment and found overall flat preference surfaces (Figure 3). This finding supports the first of two hypotheses about the shape of selection imposed by flies on purring song (preference surfaces are flat) and aligns with results from (Tinghitella et al., 2021) who investigated whether flies expressed preferences for purring songs that differed in peak frequency. The exemplars in this study span most of the tremendous variation in purring song, offering more opportunity for flies to show preferences for diverse features of purring, yet we still found no preferences. This result is not surprising considering that preference evolution often lags behind signal evolution (Broder et al., 2021; Rosenthal, 2018). Since there is a large fitness cost of not locating a host, it stands to reason that parasitoids may be selected to accept any suitable host of that species, rather than expressing strong preferences for particular individual hosts (Kruitwagen et al., 2021). Another explanation for the lack of fly preferences is the fact that purring songs are so variable (Tinghitella et al., 2018, 2021); for example, for peak frequency, ancestral song ranges from 4.8–5.2 kHz (interquartile range) while purrs show incredible variation (6.7–16.6 kHz). With such a variable signal, there would be no clear target for selection, meaning that fly preferences should not evolve for particular purring variants. This aligns with our findings; the flat fly preference surfaces we uncovered are underlain by incredible variation among flies in responses to purrs. Thus, the variation within purring song could provide additional protection from fly detection. The extreme interindividual variation among fly responses aligns with previous work that showed variation among flies in response to peak frequency variation (Tinghitella et al., 2021). It also indicates underlying genetic variation and/or plasticity among flies; there is some evidence for learning in *O. ochracea* (Paur & Gray, 2011). The relatively low response of flies to purring suggests that they are not pre‐adapted to the novel signal; however, the incredible variation in responses reveals that the capacity for preferences does exist in the population. Any preferences expressed by individual flies could have massive effects on the evolutionary trajectory of purring, considering that each gravid female may hold 57–517 planidia (larvae; Wineriter & Walker, 1990).

We can make predictions about future evolution in this system if we consider our results as well as sexual selection, as sexual and natural selection may act on signals in different ways (Gray & Cade, 1999); we previously found that female crickets have flat preference functions when exposed to purrs that vary in many dimensions (Tinghitella et al., 2021). If both natural (flies) and sexual (crickets) selection functions are flat, the new purring signal should be primarily evolving through drift, at least concerning frequency, temporal, and broadbandedness components. Future studies should track whether or not these song characteristics that do not appear to be under selection diverge in different populations. The only feature of purring songs that appears to be under selection is amplitude–natural selection should favor quiet purrs, but overall selection also depends on sexual selection upon amplitude, which remains to be tested in this system (Balenger et al., 2017).

This work expands our understanding of a rapidly evolving system but also prompts future work. For this study, we selected a population with high morph diversity (silent/flatwing, ancestral, and purring) and high fly abundance. Populations vary in cricket morph compositions and some completely lack ancestral males (Tinghitella et al., 2021), and so we may find different patterns in other populations. For instance, in populations that lack ancestral males entirely, we may expect flies to show greater selectivity among purring males. In populations that have both purring and ancestral males, we may not expect preferences to have developed for novel signals as there would be weak selection on flies to distinguish among purring songs; this could also explain the flat preference surfaces of flies in response to purring songs that we found. Future work should measure responses to novel songs in other populations while also closely monitoring cricket morph ratios and fly abundance. If we repeated this experiment in a population that lacked ancestral singers, it would also address a limitation of our study; because there were ancestral singers in our population, it is possible they could have affected fly attraction to traps, though we did not find that our ancestral traps affected fly attraction to other traps (Figure S1). Interestingly, in our lab experiments, a number of flies traveled the maximum distance (to the bottom of the cage) but failed to contact the speaker. This suggests that flies may have trouble pinpointing purrs, which may contribute to the differences we observed between field and lab experiments (more flies contacted speakers in the lab than contacted speakers in traps in the field). Future work could examine in detail how flies locate novel stimuli in the wild since fly search behavior in the wild is almost impossible to observe, necessitating that experiments take place in laboratories (e.g., Mason et al., 2005; Muller & Robert, 2001). This work provides an important baseline estimate, and future work can evaluate how preferences change over time to determine if and how flies adapt to a novel signal.

## AUTHOR CONTRIBUTIONS

**E. Dale Broder:** Conceptualization (lead); funding acquisition (supporting); investigation (equal); methodology (equal); writing – original draft (equal); writing – review and editing (lead). **James H. Gallagher:** Conceptualization (supporting); investigation (equal); methodology (equal); visualization (equal); writing – original draft (equal); writing – review and editing (equal). **Aaron W. Wikle:** Conceptualization (supporting); data curation (equal); formal analysis (lead); investigation (equal); visualization (equal); writing – review and editing (supporting). **Cameron P. Venable:** Data curation (equal); formal analysis (supporting); funding acquisition (supporting); writing – original draft (equal); writing – review and editing (supporting). **David M. Zonana:** Data curation (equal); formal analysis (equal); validation (supporting); visualization (supporting); writing – original draft (supporting); writing – review and editing (supporting). **Spencer J. Ingley:** Conceptualization (supporting); investigation (equal); project administration (equal); resources (supporting); supervision (equal); writing – original draft (supporting); writing – review and editing (supporting). **Tanner C. Smith:** Investigation (equal); methodology (supporting); supervision (supporting); writing – original draft (supporting); writing – review and editing (supporting). **Robin M. Tinghitella:** Conceptualization (lead); funding acquisition (lead); investigation (equal); methodology (equal); supervision (lead); writing – original draft (equal); writing – review and editing (equal).

## CONFLICT OF INTEREST

The authors declare no conflict of interest.

## FUNDING INFORMATION

This work and the personnel on the manuscript were supported by grants from the Ford Foundation Fellowship to CPV, the National Science Foundation to RMT (IOS 1846520 and DEB 2012041) and to DZ (PRFB2010983) and to AWW (GRFP), the American Philosophical Society to RMT and EDB, and grants from Sigma Xi, SICB, the Orthopterists' Society, DU's Shubert fund, and a Kickstarter (Frank Truslow, Bryant Palmer, Virginia Rauh, Rollin Gallagher) to JHG.

## Supporting information


Figure S1

## Data Availability

The authors declare that the data supporting the findings in this study are available within this paper and in Dryad (https://doi.org/10.5061/dryad.pzgmsbcq2). Our complete R‐markdown file is available as a supplement and on Dryad.

## References

[ece39193-bib-0001] Balenger, S. L. , Lara, L. M. , & Zuk, M. (2017). Relative amplitude of courtship song chirp and trill components does not Alter female Teleogryllus oceanicus mating behavior. Ethology: Formerly Zeitschrift Fur Tierpsychologie, 123(2), 168–173.

[ece39193-bib-0002] Bates, D. , M. Mächler , B. Bolker , Walker S 2014. Fitting linear mixed‐effects models using lme4. arXiv [stat.CO]. arXiv. http://arxiv.org/abs/1406.5823

[ece39193-bib-0003] Belwood, J. J. , & Morris, G. K. (1987). Bat predation and its influence on calling behavior in neotropical katydids. Science, 238(4823), 64–67. 17835656 10.1126/science.238.4823.64

[ece39193-bib-0004] Bertram, S. M. , Xochitl Orozco, S. , & Bellani, R. (2004). Temporal shifts in conspicuousness: Mate attraction displays of the Texas field cricket, Gryllus Texensis. Ethology: Formerly Zeitschrift Fur Tierpsychologie, 110(12), 963–975.

[ece39193-bib-0005] Broder, E. D. , Damian, O. E. , Rodríguez, R. L. , Rosenthal, G. G. , Seymoure, B. M. , & Tinghitella, R. M. (2021). Evolutionary novelty in communication between the sexes. Biology Letters, 17(2), 20200733. 33529546 10.1098/rsbl.2020.0733 PMC8086948

[ece39193-bib-0006] Cade, W. (1975). Acoustically orienting parasitoids: Fly Phonotaxis to cricket song. Science, 190(4221), 1312–1313.

[ece39193-bib-0007] Cade, W. H. , Ciceran, M. , & Murray, A. (1996). Temporal patterns of parasitoid Fly (*Ormia ochracea*) attraction to field cricket song (*Gryllus integer*). Canadian Journal of Zoology, 74(2), 393–395.

[ece39193-bib-0008] Darwin, C. (1871). The origin of species and the descent of man. Random House.

[ece39193-bib-0009] Eggleton, P. , & Belshaw, R. (1992). Insect parasitoids: An evolutionary overview. Philosophical Transactions of the Royal Society of London. Series B, Biological Sciences, 337(1279), 1–20.

[ece39193-bib-0010] Endler, J. A. (1987). Predation, light intensity and courtship behaviour in *Poecilia reticulata* (Pisces: Poeciliidae). Animal Behaviour, 35(5), 1376–1385.

[ece39193-bib-0011] Goodson, J. L. , & Adkins‐Regan, E. (1997). Playback of crows of male Japanese quail elicits female phonotaxis. The Condor, 99(4), 990–993.

[ece39193-bib-0012] Gray, D. A. , & Cade, W. H. (1999). Sex, death, and genetic variation: Natural and sexual selection on cricket song. Proceedings of the Royal Society of London. Series B: Biological Sciences, 266(1420), 707–709.

[ece39193-bib-0013] Gray, D. A. , Kunerth, H. D. , Zuk, M. , Cade, W. H. , & Balenger, S. L. (2019). Molecular biogeography and host relations of a parasitoid fly. Ecology and Evolution, 9(19), 11476–11493. 31641487 10.1002/ece3.5649 PMC6802024

[ece39193-bib-0100] Hedwig, B. , & Poulet, J. F. (2004). Complex auditory behaviour emerges from simple reactive steering. Nature, 430(7001), 781‐785. 15306810 10.1038/nature02787

[ece39193-bib-0014] Heinen‐Kay, J. L. , & Zuk, M. (2019). When does sexual signal exploitation lead to signal loss? Frontiers in Ecology and Evolution, 7, 1–11.

[ece39193-bib-0015] Kaltz, O. , & Shykoff, J. A. (1998). Local adaptation in host–parasite systems. Heredity, 81(4), 361–370.

[ece39193-bib-0016] Kruitwagen, A. , Beukeboom, L. , Wertheim, B. , & van Doorn, G. S. (2021). Evolution of parasitoid host preference and performance in response to an invasive host acting as evolutionary trap. Ecology and Evolution, 12, e9030. 10.1002/ece3.9030 PMC9251845 35813932

[ece39193-bib-0017] Lenth, R. , H. Singmann , J. Love , P. Buerkner , and M. Herve . 2019. “Estimated marginal means, aka least‐squares means. R Package Version 1.3. 2.”

[ece39193-bib-0018] Lloyd, J. E. , & Wing, S. R. (1983). Nocturnal aerial predation of fireflies by light‐seeking fireflies. Science, 222(4624), 634–635. 17843842 10.1126/science.222.4624.634

[ece39193-bib-0019] Mason, A. C. , Lee, N. , & Oshinsky, M. L. (2005). The start of phonotactic walking in the Fly *Ormia ochracea*: A kinematic study. The Journal of Experimental Biology, 208(Pt 24), 4699–4708. 16326951 10.1242/jeb.01926

[ece39193-bib-0020] Muller, P. , & Robert, D. (2001). A shot in the dark: The silent quest of a free‐flying phonotactic Fly. The Journal of Experimental Biology, 204(6), 1039–1052. 11222123 10.1242/jeb.204.6.1039

[ece39193-bib-0021] Oshinsky, M. L. , & Hoy, R. R. (2002). Physiology of the auditory afferents in an acoustic parasitoid Fly. The Journal of Neuroscience: The Official Journal of the Society for Neuroscience, 22(16), 7254–7263. 12177220 10.1523/JNEUROSCI.22-16-07254.2002 PMC6757880

[ece39193-bib-0022] Otte, D. (1994). The crickets of Hawaii: origin, systematics, and evolution (Vol. 396). The Orthopterists’ Society. Academy of Natural sciences of philadelphia.

[ece39193-bib-0023] Page, R. A. , & Ryan, M. J. (2005). Flexibility in assessment of prey cues: Frog‐eating bats and frog calls. Proceedings. Biological Sciences/The Royal Society, 272(1565), 841–847. 10.1098/rspb.2004.2998 PMC1599868 15888417

[ece39193-bib-0024] Pascoal, S. , Cezard, T. , Eik‐Nes, A. , Gharbi, K. , Majewska, J. , Payne, E. , Ritchie, M. G. , Zuk, M. , & Bailey, N. W. (2014). Rapid convergent evolution in wild crickets. Current Biology, 24(12), 1369–1374. 24881880 10.1016/j.cub.2014.04.053

[ece39193-bib-0025] Paur, J. , & Gray, D. A. (2011). Individual consistency, learning and memory in a parasitoid Fly, *Ormia ochracea* . Animal Behaviour, 82(4), 825–830.

[ece39193-bib-0026] Robert, D. , Amoroso, J. , & Hoy, R. R. (1992). The evolutionary convergence of hearing in a parasitoid Fly and its cricket host. Science, 258(5085), 1135–1137. 1439820 10.1126/science.1439820

[ece39193-bib-0027] Rosenthal, G. G. (2018). Mate choice: The evolution of sexual decision making from microbes to humans. University of California Press.

[ece39193-bib-0028] Ryan, M. J. , Fox, J. H. , Wilczynski, W. , & Rand, A. S. (1990). Sexual selection for sensory exploitation in the frog Physalaemus Pustulosus. Nature, 343(6253), 66–67. 2296291 10.1038/343066a0

[ece39193-bib-0101] Sarmiento‐Ponce, E. J. , Rogers, S. , & Hedwig, B. (2021). Does the choosiness of female crickets change as they age? Journal of Experimental Biology, 224(11), p.jeb241802. 10.1242/jeb.241802 PMC8214831 34114627

[ece39193-bib-0029] Smith, T. , Broder, E. D. , Tinghitella, R. M. , & Ingley, S. J. (2021). Using inter‐institutional collaboration to generate publishable findings through course‐based undergraduate research experiences. The American Biology Teacher, 83(7), 451–457.

[ece39193-bib-0030] Tinghitella, R. M. , Broder, E. D. , Gallagher, J. H. , Wikle, A. W. , & Zonana, D. M. (2021). Responses of intended and unintended receivers to a novel sexual signal suggest clandestine communication. Nature Communications, 12(1), 797. 10.1038/s41467-021-20971-5 PMC7862365 33542210

[ece39193-bib-0031] Tinghitella, R. M. , Broder, E. D. , Gurule‐Small, G. A. , Hallagan, C. J. , & Wilson, J. D. (2018). Purring crickets: The evolution of a novel sexual signal. The American Naturalist, 192(6), 773–782. 10.1086/700116 30444653

[ece39193-bib-0032] Vinson, S. B. (1976). Host selection by insect parasitoids. Annual Review of Entomology, 21(1), 109–133.

[ece39193-bib-0033] Wagner, W. E. (1996). Convergent song preferences between female field crickets and acoustically orienting parasitoid flies. Behavioral Ecology: Official Journal of the International Society for Behavioral Ecology, 7(3), 279–285.

[ece39193-bib-0034] Walker, T. J. (1989). A live trap for monitoring Euphasiopteryx and tests with *E. ochracea* (Diptera: Tachinidae). The Florida Entomologist, 72(2), 314–319.

[ece39193-bib-0035] Wineriter, S. A. , & Walker, T. J. (1990). Rearing phonotactic parasitoid flies [Diptera: Tachinidae, Ormiini, *Ormia* Spp.]. Entomophaga, 35(4), 621–632.

[ece39193-bib-0036] Wood, S. N. (2003). Thin plate regression splines. Journal of the Royal Statistical Society. Series B, Statistical Methodology, 65(1), 95–114.

[ece39193-bib-0037] Zuk, M. , & Kolluru, G. R. (1998). Exploitation of sexual signals by predators and parasitoids. The Quarterly Review of Biology, 73(4), 415–438.

