# Supplementary material for: Behavioral responses of a parasitoid fly to rapidly evolving host signals
Source: Ecol Evol. 2022 Aug 11;12(8):e9193. doi: 10.1002/ece3.9193 (PMC9366563; doi:10.1002/ece3.9193)
Supplement: Supplementary file 1 — Figure S1 [file ECE3-12-e9193-s001.docx]

**Supplemental Figure 1 from Broder et al. 2022**

**
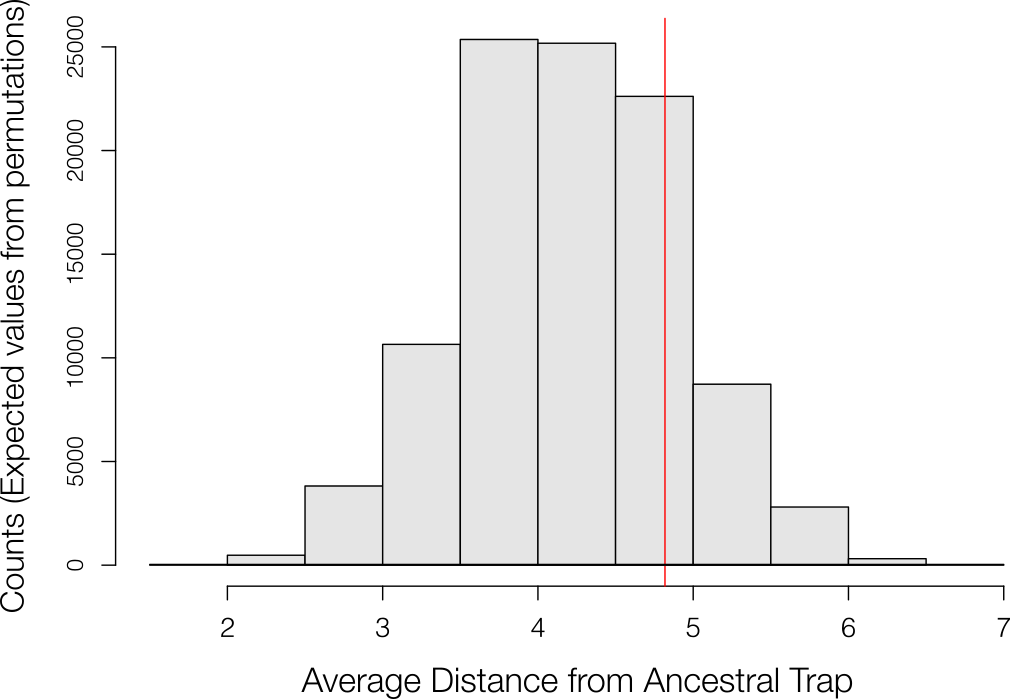
**

Traps that caught flies playing non-ancestral songs (purring N = 10, white noise N = 1) were not closer to ancestral playback speakers than expected by chance (*p* = 0.821). “Average distance from ancestral trap” on the x-axis refers to position (number of traps away) rather than linear distance; all traps were 10m apart. The red line shows the mean difference in observed trap-line position between traps capturing flies to purring or white noise stimuli and the ancestral playback speaker. Gray histogram shows the distribution of expected mean differences between ancestral and successful non-ancestral fly traps under random processes. If flies were captured at purring and white noise traps as a byproduct of their initial attraction to ancestral song, we would expect successful non-ancestral fly traps to be in closer proximity to ancestral playback speakers (smaller mean difference in trap position) than expected by chance.
